# The role of C-peptide in the attenuation of outcomes of diabetic kidney disease: a systematic review and meta-analysis

**DOI:** 10.1590/2175-8239-JBN-2017-0027

**Published:** 2018-08-13

**Authors:** Camila Marques Oliveira, Caroline Pereira Domingueti

**Affiliations:** 1 Universidade Federal de São João Del Rei, Divinópolis, MG, Brasil.

**Keywords:** C-peptide, Diabetic Nephropathy, Diabetes *Mellitus*, Peptídeo C, Nefropatias Diabéticas, Diabetes *Mellitus*

## Abstract

**Introduction:**

Preclinical trials have shown that C-peptide may contribute to the treatment of diabetic kidney disease (DKD). This systematic review and meta-analysis aimed to assess the use of C-peptide in attenuating the outcomes of DKD.

**Methods:**

Searches were made on databases PubMed, Web of Science, and Scielo for in vivo clinical and preclinical trials written in English, Portuguese or Spanish that looked into the use of C-peptide in the attenuation of the outcomes of DKD.

**Results:**

Twelve papers were included in this review, one clinical and eleven preclinical trials. In the clinical trial, DKD patients given C-peptide had lower levels of albuminuria than the subjects in the control group, but glomerular filtration rates were not significantly different. The main parameters assessed in the preclinical trials were glomerular filtration rate (six trials) and albuminuria (five trials); three trials described less hyperfiltration and three reported lower levels of albuminuria in the groups offered C-peptide. The meta-analysis revealed that the animals given C-peptide had lower glomerular volumes and lower urine potassium levels than the groups not given C-peptide.

**Conclusion:**

The results of the studies included in the systematic review diverged. However, the meta-analysis showed that the animals given C-peptide had lower glomerular volumes and lower urine potassium levels.

## RESUMO

**Introdução:**

Estudos pré-clínicos demonstraram que o peptídeo C pode contribuir para a terapia da doença renal do diabetes (DRD). Esta revisão sistemática e meta-análise teve como objetivo avaliar a utilidade do peptídeo C na atenuação dos desfechos da DRD.

**Métodos:**

Foram utilizadas as bases de dados PubMed, Web of Science e Scielo, e definidos como critérios de elegilibilidade ensaios clínicos e pré-clínicos *in vivo*, redigidos em inglês, português ou espanhol, que avaliaram a utilidade do peptídeo C na atenuação dos desfechos da DRD.

**Resultados:**

Doze artigos foram incluídos nesta revisão: onze ensaios pré-clínicos e um ensaio clínico. No ensaio clínico, os pacientes com DRD que receberam peptídeo C apresentaram menor albuminúria do que os do grupo controle, contudo não houve diferença significativa em relação à taxa de filtração glomerular. Os principais parâmetros avaliados pelos estudos pré-clínicos foram taxa de filtração glomerular (seis estudos) e albuminúria (cinco estudos), dos quais três encontraram menor hiperfiltração e três verificaram menor albuminúria no grupo que recebeu peptídeo C. A meta-análise demonstrou que os animais que receberam peptídeo C apresentaram menor volume glomerular e menor excreção urinária de potássio em comparação com aqueles que não o receberam.

**Conclusão:**

Os resultados dos estudos incluídos nesta revisão sistemática foram divergentes. Contudo, a meta-análise demonstrou que a administração do peptídeo C em animais resultou em menor volume glomerular e menor excreção urinária de potássio.

## INTRODUCTION

In the biosynthesis of insulin, pro-insulin undergoes cleavage to form insulin and C-peptide. Thirty-one amino acids are present in C-peptide. The sequence of amino acids varies between species, but the position of some amino acid residues is preserved in mammals or change by only one species.^1^ C-peptide is secreted in the bloodstream in equimolar amounts when compared to insulin and is used as an indicator of the endogenous secretion of the latter.^2^

Until recently, C-peptide was seen as an inert molecule that contributed solely to the biosynthesis of insulin,^3^ aiding in the correct folding of insulin and the formation of disulfide bridges.^1^ Multiple functional roles have been recently described for C-peptide, including binding to cell membranes, activation of signaling pathways, physiological effects, and protection against complications derived from diabetes *mellitus* (DM).^3^ Preclinical trials showed that C-peptide may improve the outcomes related to diabetic kidney disease (DKD).^4^^,^ 5

DKD - one of the microvascular complications of DM - introduces structural and functional alterations in the kidneys consequent to chronic hyperglycemia.^2^^,^ 6 Structural changes include glomerular and renal hypertrophy, thickening of the basement membrane, tubular atrophy, and interstitial fibrosis, which in turn foster the development of hyperfiltration, proteinuria, and decreased renal function.^2^ DKD is found in 20-30% of the patients with type 1 (T1DM) and type 2 (T2DM) diabetes *mellitus*; DKD negatively affects the quality of life and the survival of patients with DM.^6^ Screening for DKD includes annual urine albumin and glomerular filtration rate (GFR) tests.^7^

Some authors observed that the administration of C-peptide in diabetic rodents led to improvements in glomerulosclerosis and decreases in the thickening of the basement membrane, albuminuria, and hyperfiltration.^5^^,^ 8 ^,^ 9 Another indication of the relevance of C-peptide in the prevention of DKD stems from the outcomes of combined pancreatic islet and kidney transplantation, in which the persistent function of pancreatic islets has been associated with improved renal graft survival and function.^10^ Therefore, C-peptide has been considered a promising agent in the treatment and prevention of DKD and a possible candidate to supplement currently available therapeutic protocols, which include angiotensin-converting-enzyme inhibitors (captopril, enalapril, lisinopril etc.) and angiotensin II receptor blockers (losartan, irbesartan, telmisartan etc.).^11^ This paper aimed to present a systematic review of the literature and a meta-analysis on the use of C-peptide to attenuate the outcomes of DKD.

## METHODS

### SEARCH STRATEGY

Searches were performed on electronic databases Medline (PubMed), Web of Science, and Scielo. Medical Subject Headings (MeSH) was used to define the descriptors for the searches on databases PubMed and Web of Science, while Descritores em Ciências da Saúde (DeCS) was used to define the descriptors for the searches carried out on Scielo.

The following descriptors were used in the search for papers on PubMed and Web of Science: "C-peptide", "C Peptide", "Connecting Peptide", "Proinsulin C-Peptide", "Proinsulin C Peptide", "C-Peptide, Proinsulin", "C Peptide, Proinsulin", combined with descriptors "diabetic nephropathies", "Nephropathies, Diabetic", "Nephropathy, Diabetic", "Diabetic Nephropathy", "Diabetic Kidney Disease", "Diabetic Kidney Diseases", "Kidney Disease, Diabetic", "Kidney Diseases, Diabetic", "Diabetic Glomerulosclerosis", "Kimmelstiel-Wilson Syndrome", "Kimmelstiel Wilson Syndrome", "Syndrome, Kimmelstiel-Wilson", "Kimmelstiel-Wilson Disease", "Kimmelstiel Wilson Disease", "Nodular Glomerulosclerosis", "Glomerulosclerosis, Nodular", "Glomerulosclerosis, Diabetic", "Intracapillary Glomerulosclerosis", with connector "AND" between the terms. The selection of papers from Scielo used descriptors "C-peptide" and "proinsulin C-peptide", combined with descriptors "diabetic nephropathies", "glomerulosclerosis, diabetic", "diabetic glomerulosclerosis", with connector "AND" between the terms (Annex 1).

### ELIGIBILITY CRITERIA

Searches were made on databases PubMed, Web of Science, and Scielo for in vivo clinical and preclinical trials written in English, Portuguese or Spanish that looked into the use of C-peptide in the attenuation of the outcomes of DKD. The following eligibility criteria were established in accordance with the PRISMA^12^ recommendations:

- Population: Animals given only C-peptide or humans given hypoglycemic drugs and C-peptide.
- Exposure: Administration of C-peptide.
- Control: Animals given saline solution only or humans given hypoglycemic drugs and saline solution.
- Outcome: Attenuation of DKD outcomes.
- Study design: In vivo preclinical or clinical trial.

The results from the comparisons made between animals offered interventions other than the administration of C-peptide (insulin or drug therapy) were not included in the systematic review. Review papers, in vitro trials, and case reports were excluded. No restrictions were applied to the period of publication of the papers included in the review. Database searches were carried out by June 2017.

### PAPER SELECTION

Two individuals selected the papers independently in two stages. Differences of opinion were discussed until the two individuals reached agreement. In the first stage, the papers were identified based on the search criteria and duplicates were excluded. Then the titles and abstracts were read so that only papers meeting the eligibility criteria were included. In the second stage, the papers selected in the first stage were read and the ones meeting the eligibility criteria were included in the systematic review.

### EXTRACTION OF DATA FROM THE SELECTED PAPERS

The following data were extracted from the selected preclinical trials: strain of rodents used in the study; method used to induce DM; time of exposure to DM; type of C-peptide; C-peptide dose, route, site, and time of administration; size of the case and control groups; parameters used to characterize DKD; and outcomes. Two individuals independently extracted data from the papers and possible differences of opinion were discussed until an agreement was reached. The following data were extracted from the clinical trial: type of DM; C-peptide dose and time of administration; size of the case and control groups; parameters used to characterize DKD; and outcomes.

### METHODOLOGICAL QUALITY OF THE INCLUDED PAPERS

Two individuals independently assessed the methodological quality of the preclinical trials included in the review and resolved possible differences of opinion until an agreement was reached. SYRCLE^13^ was used to assess the risk of bias in animal studies. The tool covers the following categories: selection bias, performance bias, detection bias, attrition bias, reporting bias, and other sources of bias. Ten questions were used to assess the papers included in the systematic review; questions answered with a YES meant low risk of bias; questions answered with a NO suggested high risk of bias; questions answered with UNCLEAR indicated unclear risk of bias. It is not recommended to calculate the summation of scores of each individual study using this tool.^13^

### META-ANALYSIS

The meta-analysis included only preclinical trials looking into the same outcomes, using the same method to induce DM in the animals, presenting results in the form of mean values and standard deviations, and using measurement units reciprocally convertible into one another. Studies looking at outcomes glomerular volume and GFR were grouped based on the time of exposure to diabetes in the meta-analysis, since longer duration of exposure might lead to greater renal involvement.^11^ The mean values, standard deviations, and sample sizes of the groups given C-peptide and of the control groups in each study were considered in the meta-analysis; heterogeneity between studies was assessed with the I-square test. Studies with I^2^ > 50% and *p*-value < 0.10 were deemed heterogeneous. The differences between mean values were calculated with a random effects model for heterogeneous studies and with a fixed effects model for homogeneous studies. Statistical package *Review Manager* version 5 was used in the meta-analysis.

## RESULTS

**Figure 1:**
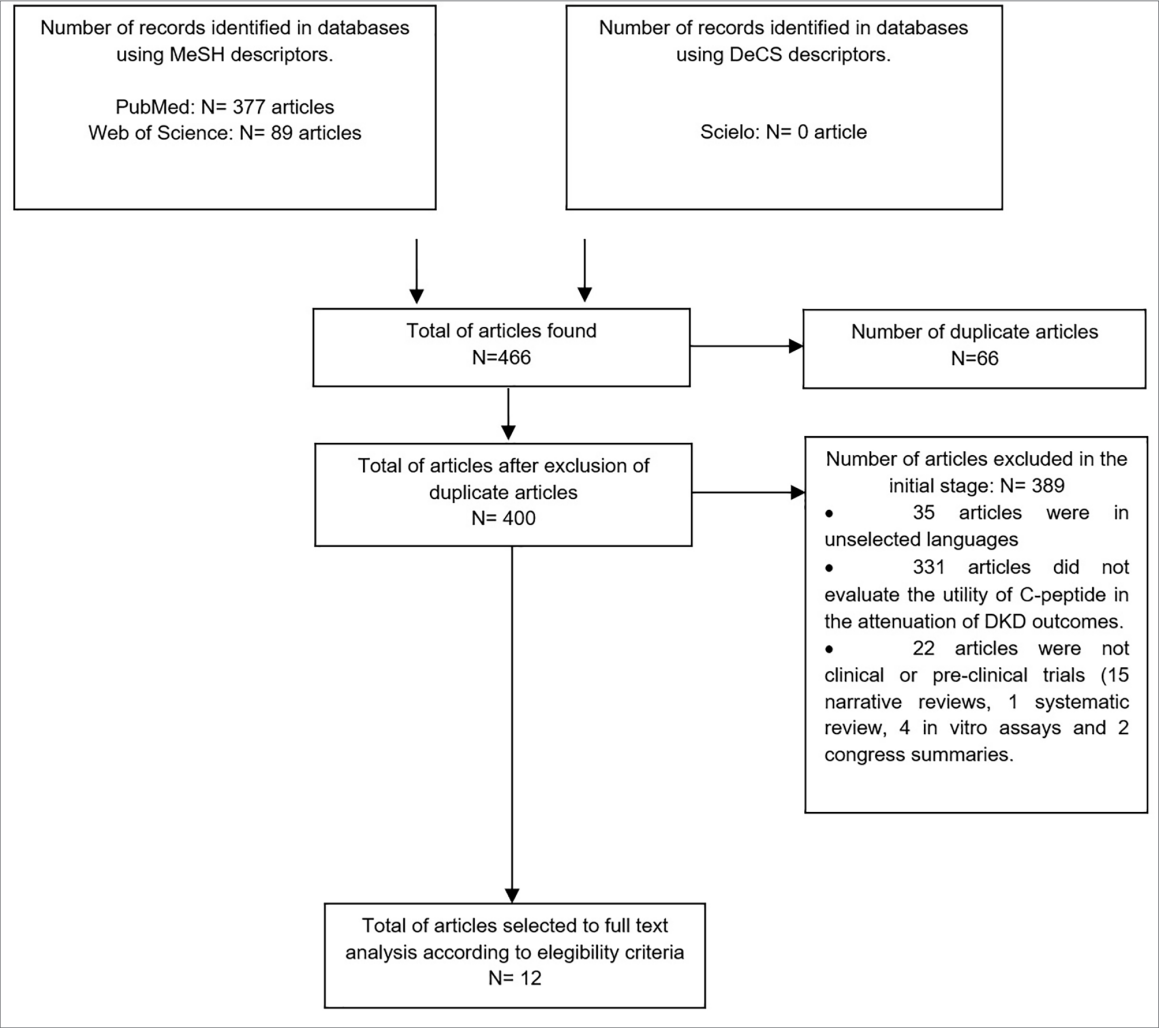
Flowchart showing the process by which papers were selected for the systematic review based on the defined eligibility criteria.

The schematic diagram presented on Figure 1 shows the paper selection stages used in the systematic review. Twelve papers were included in the review based on the eligibility criteria.

Only one clinical trial on the effects of administering C-peptide combined with insulin to patients with T1DM and DKD for six months came out from the search for papers.^14^ DKD was defined by the presence of urine albumin between 20 and 300 µg/min. The age of the patients with DKD ranged from 20 to 30 years. The group given C-peptide included ten patients, while the group not given C-peptide had eleven patients. Human C-peptide was administered subcutaneously in the abdomen of the subjects at a dosage of 600 nmol (divided in three doses within 24 hours) for three months. The group given C-peptide had lower urine albumin levels (µg/min) after two (*p* < 0.05) and three months (*p* < 0,01) of treatment and lower urine albumin-to-creatinine ratios after three months of treatment (*p* < 0.01) when compared to controls. No statistically significant differences were found between the groups for GFR.

The characteristics of the eleven included preclinical trials are shown in Table 1. The studies were published between the years of 2001 and 2015; seven^9^^,^ 16 ^,^ 17 ^,^ 18 ^,^ 19 ^,^ 21 ^,^ 22 (64%) used Sprague-Dawley rats, two^15^^,^ 20 (18%) used Wistar rats, one^8^ (9%) used C57/BI6J mice, and one^5^ (9%) used Goto-Kakizaki rats.

**Table 1:** Characteristics of the selected studies

Author/Year	Rodent strain/time of exposure to diabetes	Method to induce DM	Type of C-peptide administered	Dose of C-peptide administered/route/site and time of administration	Size of the case and control groups	Assessed parameters
Xu*et al.*, 2015^5^	Goto-Gakizaki rats/10 weeks	Fat-rich diet for two weeks	Rat C-peptide	50 pmol/kg/min of C-peptide administered subcutaneously for 12 weeks through a micropump implanted in the abdominal cavity	Group with DM given C-peptide = 8; group with DM not given C-peptide = 8	Albuminuria, fibronectin synthesis expression
Nakamoto *et al.,* 2015^15^	Wistar rats/17 to 18 weeks	Streptozotocin 50 mg/kg	Not reported	50 pmol/kg/min of C-peptide administered subcutaneously for 24 hours with a micropump implanted on the back of the rats	Group with DM given C-peptide = 20; group with DM not given C-peptide = 11	Space between podocyte foot processes
Sun*et al.,* 2010^16^	Sprague-Dawley rats/2 weeks	Intravenous injection of streptozotocin 50 mg/kg	Human C-peptide	130 nmol/kg of C-peptide injected subcutaneously twice a day for 8 weeks	Group with DM given C-peptide = 9; group with DM not given C-peptide = 9	Kidney-to-body weight ratio, glomerular volume, ratio of the extracellular matrix area to the whole glomerular area
Kamikawa *et al.,* 2008^17^	Sprague-Dawley rats/1 week	Intraperitoneal injection of streptozotocin 45 mg/kg	Human C-peptide	35 pmol/min/kg of C-peptide administered subcutaneously for a week through an osmotic pump	Group with DM given C-peptide = 7; group with DM not given C-peptide = 11	Expression of eNOS in the kidneys and glomerular volume
Nordquist *et al.,* 2007^18^	Sprague-Dawley rats/2 weeks	Intravenous injection of streptozotocin 55 mg/kg	Rat C-peptide and C-peptide fragment	50 pmol/kg/min of C-peptide and 500 pmol/kg/min of C-peptide fragment administered for 100 minutes	Group with DM given C-peptide = 9; Group with DM given C-peptide fragment = 9; group with DM not given C-peptide = 8	GFR calculated based on inulin clearance, urinary flow rate, urine sodium and potassium
Rebsomen *et al*., 2006^19^	Sprague-Dawley rats/1 week	Intraperitoneal injection of streptozotocin 65 mg/kg	Rat C-peptide	50 pmol/kg/dia of C-peptide administered for 28 days by continuous intraperitoneal infusion with an osmotic minipump placed in the peritoneal cavity	Group with DM given C-peptide = 6; group with DM not given C-peptide = 6	Proteinuria, urine sodium, and GFR calculated based on creatinine clearance
Maezawa *et al.,* 2006^8^	C57/Bl6J mice/2 weeks	Intraperitoneal injection of streptozotocin 100 mg/kg	Rat C-peptide and modified C-peptide	290 pmol/kg/min of C-peptide administered subcutaneously for 24 hours or a week with an osmotic pump	Group with DM given C-peptide = 7; group with DM given modified C-peptide = 7; group with DM not given C-peptide = 7	Albuminuria, expression of collagen IV and TFG-β, and GFR calculated based on creatinine clearance
Samnegard *et al.,* 2005^20^	Wistar rats/4 weeks	Intravenous injection of streptozotocin 60 mg/kg	Rat C-peptide	50 pmol/kg/min of C-peptide administered for four weeks by continuous intraperitoneal infusion with an osmotic pump placed on the neck	Group with DM given C-peptide = 11; group with DM not given C-peptide = 11	Glomerular volume, mesangial volume, mesangial matrix volume, albuminuria, urine potassium and sodium, GFR calculated based on inulin clearance, and glomerular basement membrane thickening
Samnegard *et al.*, 2004^21^	Sprague-Dawley rats/2 weeks	Intravenous injection of streptozotocin 55 mg/kg	Rat C-peptide	50 pmol/kg/min of C-peptide administered for 60 minutes	Group with DM given C-peptide = 13; group with DM not given C-peptide = 7	GFR calculated based on inulin clearance
Huang *et al.*, 2002^22^	Sprague-Dawley rats/2 weeks	Administration of streptozotocin 65 mg/kg	Human C-peptide	Intravenous bolus injection of 0,6; 1.8; 6; 18 and 60 nmol/kg followed by continuous infusion of 30 nmol/kg/h of C-peptide	Group with DM given C-peptide = 11 (dose 0.1x); 10 (dose 0.3x); 7 (dose 1x); 9 (dose 3x); 8 (dose 10x); group with DM not given C-peptide = 14	Albuminuria and serum calcium and sodium levels
Samnegard *et al*., 2001^9^	Sprague-Dawley rats/2 weeks	Intravenous injection of streptozotocin 60 mg/kg	Rat C-peptide	50 pmol/kg/min of C-peptide administered for 2 weeks by continuous intravenous infusion with an osmotic pump placed in the subcutaneous tissue of the neck connected to a catheter inserted in the right jugular vein of the rats	Group with DM given C-peptide = 7; group with DM not given C-peptide = 7	Glomerular volume, GFR calculated based on inulin clearance, albuminuria, and urine sodium and potassium

DM - Diabetes *mellitus*; eNOS - endothelial nitric oxide synthase expression; GFR - glomerular filtration rate; TGF-β - transforming growth factor beta

Ten (91%) studies induced DM by administering streptozotocin; five^9^^,^ 16 ^,^ 18 ^,^ 20 ^,^ 21 (45%) used the intravenous route; three^8^^,^ 17 ^,^ 19 (27%) used the intraperitoneal route of administration; and two^15^^,^ 22 (18%) did not report the route of administration. Only one^5^ (9%) induced DM by means of a fat-rich diet for two weeks. The dose administered ranged from 45 to 100 mg/kg. The time of exposure to DM was two weeks in six^8^^,^ 9 ^,^ 16 ^,^ 18 ^,^ 21 ^,^ 22 studies (55%), one week in two^17^^,^ 19 studies (18%), four weeks in one^20^ study (9%), ten weeks in one^5^ study (9%), and 17 to 18 weeks in one^15^ study (9%).

In terms of the type of C-peptide given to the case groups, five 5 ^,^ 9 ^,^ 19 ^,^ 20 ^,^ 21 (45%) studies administered only rat C-peptide, three^16^^,^ 17 ^,^ 22 (27%) administered only human C-peptide, one^15^ (9%) did not report the type of C-peptide, one (9%)^18^ administered rat C-peptide and C-peptide fragment, and one^8^ (9%) administered rat C-peptide and modified C-peptide. In relation to the route of administration, six^8^^,^ 15 ^,^ 16 ^,^ 17 ^,^ 20 (55%) studies administered C-peptide subcutaneously, two^18^^,^ 21 (18%) did not report the route of administration, two^9^^,^ 22 (18%) administered C-peptide intravenously, and one^19^ (9%) administered via the intraperitoneal route. The doses administered ranged from 35 pmol/kg/min to 130 nmol/kg/min; the time of administration varied from 60 minutes to 12 weeks in the selected studies. The size of the samples included in the studies ranged from six to 20 subjects in the case group and from six to 14 subjects in the control group.

Table 1 shows the parameters assessed in each of the studies. Six^8^^,^ 9 ^,^ 18 ^,^ 19 ^,^ 20 ^,^ 21 (55%) studies analyzed the GFR, five^5^^,^ 8 ^,^ 9 ^,^ 20 ^,^ 22 (45%) looked at urine albumin, four^9^^,^ 16 ^,^ 17 ^,^ 20 (36%) assessed glomerular volume, and three^9^^,^ 18 ^,^ 20 (27%) considered urine sodium and potassium.

The results of the preclinical studies are shown in Table 2. Three^9^^,^ 18 ^,^ 21 studies reported less hyperfiltration in the groups given C-peptide than in controls; three^5^^,^ 8 ^,^ 9 found lower urine albumin levels in the groups given C-peptide (although not in the group given modified C-peptide); three^9^^,^ 15 ^,^ 19 studies found lower glomerular volumes in the groups given C-peptide; and none of the studies looking at urine sodium and potassium found significant differences between groups.

**Table 2:** Outcomes from selected studies

Author/Year	Outcomes
Xu *et al.*, 2015^5^	Group given C-peptide had lower albuminuria (*p* < 0.01) and fibronectin expression (*p* < 0.01) than controls.
Nakamoto *et al.*, 2015^15^	Group given C-peptide had wider spaces between podocyte foot processes than controls (*p* < 0.05).
Sun *et al.*, 2010^16^	Group given C-peptide had lower kidney-to-body weight ratio (*p* < 0.05), lower glomerular volume (p < 0.01), and lower ratio of the extracellular matrix area to the whole glomerular area (*p* < 0.01) than controls.
Kamikawa *et al*., 2008^17^	Group given C-peptide had lower kidney eNOS expression than controls (*p* < 0.05); no significant differences were seen between the group given C-peptide and controls in terms of glomerular volume.
Nordquist *et al*., 2007^18^	Group given C-peptide and C-peptide fragment had less hyperfiltration than controls (*p* < 0.05); no significant differences were seen between the groups given C-peptide or C-peptide fragment and controls in relation to urinary flow rate and urine sodium and potassium.
Rebsomen *et al*., 2006^19^	Group given C-peptide had lower proteinuria and urine sodium than controls (*p* < 0.05); no significant differences were seen between the group given C-peptide and controls in regards to GFR.
Maezawa*et al.*, 2006^8^	Group given C-peptide had lower albuminuria (*p* < 0.01) and expression of collagen IV and TGF-β (*p* < 0.05) than controls; no significant differences were seen between the group given modified C-peptide and controls in regards to albuminuria and expression of collagen IV and TGF-β; no significant differences were seen between the groups in terms of GFR.
Samnegard *et al.*, 2005^20^	Group given C-peptide had lower glomerular volume (*p* < 0,001), lower mesangial volume (*p* < 0.01), and lower mesangial matrix volume (*p* < 0.01) than controls; no significant differences were seen between the group given C-peptide and controls in regards to albuminuria, urine sodium and potassium, GFR, and thickening of the glomerular basement membrane.
Samnegard *et al.*, 2004^21^	Group given C-peptide had less hyperfiltration than controls.
Huang *et al.*, 2002^22^	No significant differences were seen between the group given C-peptide and controls in regards to albuminuria and serum sodium and potassium levels.
Samnegard *et al.*, 2001^9^	Group given C-peptide had lower glomerular volume, less hyperfiltration (*p* < 0.001) and albuminuria than controls; no significant differences were seen between the group given C-peptide and controls in regards to urine sodium and potassium.

Table 3 shows the findings on methodological quality of the preclinical trials. All studies were found to be at low risk of bias.

**Table 3:** Quality assessment of the studies according to the SYRCLE scale

Author/Year	Selection bias			Performance bias		Detection bias		Attrition bias	Reporting bias	Other sources of bias
	1	2	3	4	5	6	7	8	9	10
Xu *et al*., 2015^5^	S	Y	?	Y	?	?	?	Y	Y	Y
Nakamoto *et al.*, 2015^15^	Y	Y	?	Y	?	?	?	Y	Y	Y
Sun *et al.*, 2010^16^	Y	Y	?	Y	?	?	?	Y	Y	Y
Kamikawa *et al.*, 2008^17^	Y	Y	?	Y	?	?	?	N	Y	Y
Nordquist *et al*., 2007^18^	Y	Y	?	Y	?	?	?	Y	Y	Y
Rebsomen *et al.*, 2006^19^	Y	Y	?	Y	?	?	?	Y	Y	Y
Maezawa *et al.*, 2006^8^	Y	Y	?	Y	?	?	?	Y	Y	Y
Samnegard *et al.*, 2005^20^	Y	Y	?	Y	?	?	?	Y	Y	Y
Samnegard *et al.*, 2004^21^	Y	Y	?	Y	?	?	?	Y	Y	Y
Huang *et al.*, 2002^22^	Y	Y	?	Y	?	?	?	Y	Y	Y
Samnegard *et al.*, 2001^9^	Y	Y	?	Y	?	?	?	Y	Y	Y

Y - YES (low risk of bias); N - NO (high risk of bias); ? - unclear (unclear risk of bias); 1- Sequence generation: the subjects in all studies were randomly assigned to the case or control groups; 2- Baseline characteristics: the case and control groups in all studies were given streptozotocin or fat-rich diet and developed DM; therefore, both had DM at the start of the trial; 3 - Allocation concealment: none of the papers described the use of allocation concealment in the distribution of subjects between the case and control groups; 4 - Random housing: case and control subjects were randomly assigned to their housing units and were exposed to equal conditions; 5 - Blinding: none of the papers reported whether researchers were aware of which animals were given the prescribed interventions (saline solution or C-peptide); 6 - Random outcome assessment: none of the papers established whether the outcomes of case and control groups were assessed randomly; 7 - Blinding: none of the papers described whether the researchers were aware of which animals had been assigned to which intervention (saline solution or C-peptide) in the assessment of outcomes; 8 - Incomplete outcome data: None of the papers but one excluded animals in the assessment of outcomes; 9 - Selective outcome reporting: none of the studies selectively reported outcomes with significant results; 10 - Other sources of bias: none of the papers had other sources of bias.

**Figure 2:**
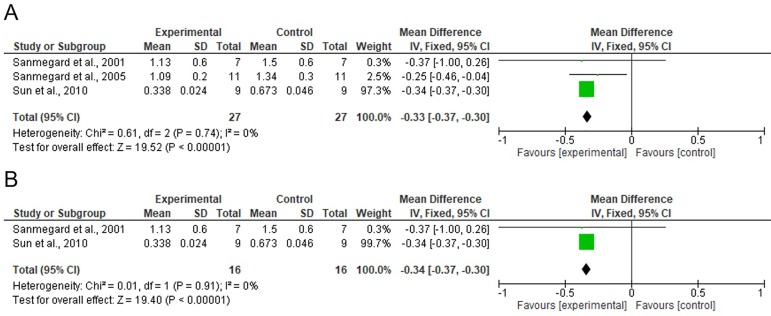
Meta-analysis of the studies assessing glomerular volume: A) studies with different times of exposure to diabetes (two or four weeks); B) studies with equal times of exposure to diabetes (two weeks). Mean values, standard deviations, differences between mean values, and 95% confidence interval were raised to 10.^6^

**Figure 3:**
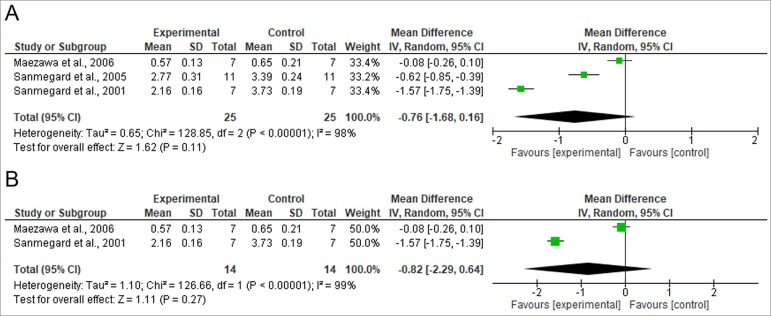
Meta-analysis of the studies assessing glomerular filtration rate: studies with different times of exposure to diabetes (two or four weeks); B) studies with equal times of exposure to diabetes (two weeks).

**Figure 4:**
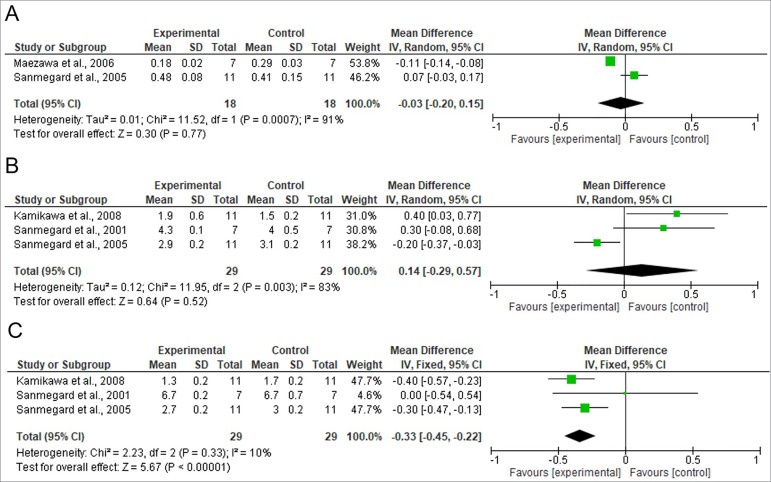
Meta-analysis of the studies assessing A) albuminuria; B) urinary excretion of sodium; C) urinary excretion of potassium.

Figures 2, 3, and 4 show the results from the meta-analysis. The studies looking at glomerular volume and urine potassium included in the meta-analysis were categorized as homogeneous (I^2^ = 0% for studies looking at glomerular volume with different times of exposure to DM and equal times of exposure to DM, and I^2^ = 10% for studies analyzing urine potassium). The studies considered in the meta-analysis in which GFR, albuminuria, and urine sodium were analyzed, were categorized as heterogeneous (I^2^ = 98% and *p* < 0.001, and I^2^ = 99% and *p* < 0.001 for the studies looking at GFR with different times of exposure to DM and equal times of exposure to DM, respectively; I^2^ = 91% and *p* < 0.001 and I^2^ = 83% and *p* = 0.003 for studies analyzing albuminuria and urine sodium, respectively).

The meta-analysis revealed that the animals given C-peptide had significantly decreased glomerular volumes when compared to subjects not given C-peptide with different times of exposure to DM [difference between mean values = -0.33 x 10^6^ (-0.37 x 10^6^ - -0.30 x 10^6^), *p* < 0.001] and equal times of exposure to DM [difference between mean values = -0.34 x 10^6^ (-0.37 x 10^6^ - -0.30 x 10^6^), *p* < 0.001]. The meta-analysis also found that the animals given C-peptide had significantly lower urine potassium levels than the subjects not given C-peptide [difference between mean values = -0.33 (-0.45 - -0.22), *p* < 0.001]. In regards to the GFR, no significant differences were found between animals given C-peptide and animals not given C-peptide in studies with different times of exposure to DM [difference between mean values = -0.76 (-1.68 - 0.16), *p* = 0.11] or in equal times of exposure to DM [difference between mean values = -0.82 (-2.29 - 0.64), *p* = 0.27]. No significant differences were found between groups in relation to albuminuria and urine sodium [difference between mean values = -0.03 (-0.20 - 0.15), *p* = 0.77; 0.14 (-0.29 - 0.57, *p* = 0.52, respectively].

## DISCUSSION

This study aimed to assess the use of C-peptide to attenuate the effects of DKD by means of a systematic review of the literature and a meta-analysis.

Only one^14^ clinical trial was included in the systematic review, in which subjects with T1DM and DKD given C-peptide had lower albuminuria levels after three months of treatment in comparison with controls. However, no significant differences were seen between groups in regards to the GFR. Therefore, one might conclude that the administration of C-peptide to subjects with T1DM and DKD may help decrease urine albumin levels and, therefore, improve DKD.

GFR and urine albumin are the main parameters used in the assessment of DKD in humans.^23^ Six of the preclinical trials included in this review analyzed the GFR and five looked at urine albumin; three found less hyperfiltration and three reported lower urine albumin levels in the groups given C-peptide (although not in the group given modified C-peptide). However, no significant differences were found between the animals given C-peptide and the ones not given C-peptide in regards to GFR and urine albumin in the meta-analysis. A possible explanation for this finding is the fact that the studies used different methods to determine the GFR and urine albumin. Two^9^^,^ 20 of the studies in the meta-analysis calculated the GFR based on inulin clearance and one^8^ having creatinine clearance as a reference. One^8^ study calculated albuminuria based on ELISA test results and another^20^ resorted to nephelometry.

Two possible mechanisms might explain the decrease in hyperfiltration and urine albumin in diabetic rodents after the administration of C-peptide. C-peptide constricts the afferent arteriole in the glomerulus at the same time as the efferent arteriole dilates, thus decreasing the glomerular filtration pressure, the GFR, and urine albumin without changing renal blood flow.^24^ The constriction of the afferent arteriole by C-peptide might be related to the expression of eNOS promoted by C-peptide.^17^ C-peptide also blocks the activity of renal Na^+^-K^+^-ATPase (increased with the onset of DM), thus decreasing the reabsorption of Na^+^ by the proximal convoluted tubule (increasing the urinary excretion of sodium), the glomerular filtration pressure and, thus, the GFR and urine albumin.^24^

Another possible mechanism for the decrease in urine albumin is based on the fact that C-peptide makes the glomerular filtration barrier less permeable (thus protecting its integrity), since C-peptide prevents decreases in the expression of podocin in the renal glomeruli. This mechanism may also be associated with the lower levels of proteinuria seen in the animals given C-peptide.^25^

Four studies looked at glomerular volume, and three reported lower volumes in the groups given C-peptide, a finding confirmed in the meta-analysis when studies with different times of exposure to DM (two and four weeks) and equal times of exposure to DM (two weeks) were considered. None of the three studies assessing urine sodium and potassium reported significant differences between groups. However, the meta-analysis revealed that the animals given C-peptide had significantly lower urinary excretion of potassium than the animals not given C-peptide.

Parameters space between the foot processes; kidney-to-body weight ratio; ratio of the extracellular matrix area to the whole glomerular area; renal expression of endothelial nitric oxide synthase (eNOS); urinary flow rate; proteinuria; urinary excretion of sodium; expression of fibronectin, collagen IV and transforming growth factor-β (TGF-β); mesangial volume; mesangial matrix volume; thickening of the glomerular basement membrane; and sodium and potassium serum levels were each assessed in only one study.

The groups given C-peptide had lower kidney-to-body weight ratios; lower ratios of the extracellular matrix area to the whole glomerular area; decreased renal expression of eNOS; less proteinuria; lower urinary excretion of sodium; lower expression of fibronectin, collagen IV, TGF-β; lower mesangial volumes; and lower mesangial matrix volumes (although the group given modified C-peptide did not have lower expression of collagen IV or TGF-β) in relation to controls. A possible explanation for these findings revolves around the fact that C-peptide suppresses the exacerbated synthesis of extracellular matrix components (collagen IV in particular), thus preventing component accumulation, impeding the thickening of the glomerular basement membrane, precluding the expansion of the mesangial matrix, and consequently preventing glomerular hypertrophy.^16^ C-peptide also inhibits the expression of TGF-β, an inducer of the synthesis of extracellular matrix components, possibly contributing to lower synthesis of collagen IV and fibronectin.^8^

The studies included in this review used Wistar rats, Sprague-Dawley rats, Goto-Kakizaki rats, and C57/Bl6J mice. Wistars and Sprague-Dawley rats, the two most commonly used species in laboratory experiments,^26^ were also the most commonly used in the studies included in this review. In most of the studies, DM was induced with the administration of streptozotocin, a glucosamine-nitrosourea compound used to produce DM in experimental animals^27^ by obliterating their pancreatic beta cells.^28^ Only one study induced T2DM by offering the animals a fat-rich diet.^5^

The animals in the studies were given human C-peptide, rat C-peptide, C-peptide fragment or modified C-peptide. There is considerable equivalence between rat and human C-peptide, with both having 31 amino acids in their structures, although higher levels of human C-peptide in relation to rat C-peptide are required to produce effects in rats, possibly on account of the different disposition of amino acids in the two compounds.^16^ C-peptide fragment, formed by the rat C-peptide carboxy terminal pentapeptide (EVARQ), produces effects similar to the whole peptide and indicates the site of activity of C-peptide;^29^ modified C-peptide features the same amino acids seen in C-peptide, however randomly organized.^8^ Modified C-peptide had no effect in the study in which it was used.

The type, dose, and time of administration of C-peptide, the time of exposure to DM, and the strain of rodents varied between studies, which possibly led to differences in the reported results. None of the studies reported procedures to calculate the sample size. However, considering that a difference of 20% was seen between the mean values reported for case and control groups along with a coefficient of variation of 15%, five animals per group would be needed to reach a significance of 0.05,^30^ indicating that the size of the samples was adequate in all studies included in the systematic review.

The preclinical trials included in the systematic review may be categorized as having low risk of bias according to the criteria of the SYRCLE scale. Nonetheless, the review has its limitations. The selected studies considered different parameters, and only some of the studies looked into GFR and albuminuria - the main parameters used in the assessment of DKD in medical practice. Some studies did not report the values for the analyzed parameters and only mentioned whether the results were significantly different, which made it impossible to use them in the meta-analysis. Only one clinical trial was included, a fact that hampered the assessment of the role of C-peptide in the attenuation of the outcomes of DKD in humans. Moreover, the non-inclusion of other databases in the search for papers and publication bias (papers showing negative results are often not published or are published in journals not indexed with the selected databases) may compromise the generalization of the results found in the review.

In addition to being few, the studies included in the meta-analysis in which GFR, albuminuria, and urine sodium were assessed, were significantly heterogeneous in relation to each other, a factor that may have compromised the outcome of the meta-analysis. Heterogeneity derives from the different designs adopted in the included studies and the choices made for parameters such as the type of animal used, method used to calculate the GFR and albuminuria, and dose and time of administration of C-peptide. This observation indicates the need for further standardization of future preclinical trials devised to assess the use of C-peptide in the attenuation of the outcomes of DKD.

A previously published systematic review and meta-analysis^31^ looked into the therapeutic use of C-peptide in kidney disease. The study observed that there were decreases in proteinuria, glomerular volume, and GFR in animals with DM given C-peptide in relation to animals not given C-peptide. The results of the present meta-analysis also showed decreases in the glomerular volume of the animals treated with C-peptide. These findings were seen in the studies with different times of exposure (two and four weeks) to DM and equal times of exposure (two weeks) to DM, indicating that the administration of C-peptide may be effective when performed at different times throughout the progression of the disease in animals.

Despite the conflicting results published in the studies included in this systematic review, the meta-analysis showed that the animals given C-peptide had lower glomerular volumes and lower levels of urine potassium when compared to subjects not given C-peptide, indicating that C-peptide may help attenuate the progression of DKD. However, more preclinical and clinical trials are required to further assess the possible clinical uses of C-peptide in T1DM and T2DM.

## CONCLUSION

The results of the studies included in this systematic review diverged. However, the meta-analysis showed that the administration of C-peptide led to lower glomerular volumes and lower levels of urine potassium.

## SUPPLEMENTARY MATERIAL

The following document is available online:

Annex 1
