# Supplementary material for: The role of C-peptide in the attenuation of outcomes of diabetic kidney disease: a systematic review and meta-analysis
Source: J Bras Nefrol. 2018 Aug 13;40(4):375–87. doi: 10.1590/2175-8239-JBN-2017-0027 (PMC6533998; doi:10.1590/2175-8239-JBN-2017-0027)
Supplement: Supplementary file 1 [file 2175-8239-jbn-2017-0027-suppl01.pdf]

Supplementary Material to " The role of C-peptide in the attenuation of outcomes of diabetic kidney disease: a systematic review and meta-analysis"

Annex 1

The following search structures were used to find papers on databases PubMed and Web of Science:

(“C-peptide” or “C Peptide” or “Connecting Peptide” or “Proinsulin C-Peptide” or “Proinsulin C Peptide” or “C-Peptide, Proinsulin” or “C Peptide, Proinsulin”) and (“diabetic nephropathies” or “Nephropathies, Diabetic” or “Nephropathy, Diabetic” or “Diabetic Nephropathy” or “Diabetic Kidney Disease” or “Diabetic Kidney Diseases” or “Kidney Disease, Diabetic” or “Kidney Diseases, Diabetic” or “Diabetic Glomerulosclerosis” or “Kimmelstiel-Wilson Syndrome” or “Kimmelstiel Wilson Syndrome” or “Syndrome, Kimmelstiel-Wilson” or “Kimmelstiel-Wilson Disease” or “Kimmelstiel Wilson Disease” or “Nodular Glomerulosclerosis” or “Glomerulosclerosis, Nodular” or “Glomerulosclerosis, Diabetic” or “Intracapillary Glomerulosclerosis”).

The following search structures were used to find papers on database SciELO:

(“C-peptide” or “proinsulin C-peptide”) and (“diabetic nephropathies” or “glomerulosclerosis, diabetic” or “diabetic glomerulosclerosis”).
